# The Impact on Ovarian Reserve of Different Hemostasis Methods in Laparoscopic Cystectomy: A Systematic Review and Meta-analysis

**DOI:** 10.1055/s-0039-1692697

**Published:** 2019-06

**Authors:** Clara Micalli Ferruzzi Baracat, Helizabet Salomão Ayroza Abdalla-Ribeiro, Raquel Silveira da Cunha Araujo, Wanderley Marques Bernando, Paulo Ayroza Ribeiro

**Affiliations:** 1 Irmandade da Santa Casa de Misericórdia de São Paulo, São Paulo, SP, Brazil; 2 Hospital das Clínicas, Faculdade de Medicina, Universidade de São Paulo, São Paulo, SP, Brazil

**Keywords:** laparoscopy, ovarian reserve, ovarian cyst, systematic review, meta-analysis, laparoscopia, reserva ovariana, cisto ovariano, revisão sistemática, meta-análise

## Abstract

**Objective** The objective of this review was to analyze the impact on ovarian reserve of the different hemostatic methods used during laparoscopic cystectomy.

**Data Sources** The studies were identified by searching electronic databases (MEDLINE, Embase, Cochrane, LILACS) and scanning reference lists of articles.

**Methods of Study Selection** We selected clinical trials that assessed the influence of hemostatic techniques on ovarian reserve in patients with ovarian cysts with benign sonographic appearance submitted to laparoscopic cystectomy by stripping technique. The included trials compared different laparoscopic hemostatic techniques: suture, bipolar electrocoagulation, ultrasonic energy and hemostatic sealants. The outcomes evaluated were level of serum anti-Mullerian hormone (AMH) and antral follicle count (AFC). The possibility of publication bias was evaluated by funnel plots.

**Tabulation, Integration and Results** Twelve trials involving 1,047 patients were evaluated. Laparoscopic suture was superior to bipolar coagulation when evaluating serum AMH and AFC, in the 1st, 3rd, 6th and 12th month after surgery. In the comparison between bipolar and hemostatic sealants, the results favored the use of hemostatic agents. The use of ultrasonic energy was not superior to the use of bipolar energy.

**Conclusion** We recommend suture for hemostasis during laparoscopic cystectomy.

## Abstract (pt)

**Resumo:**

**Objetivo** O objetivo desta revisão foi comparar o impacto dos diferentes métodos hemostáticos na reserva ovariana durante a ooforoplastia laparoscópica.

**Fontes de Dados** Os estudos foram identificados através da pesquisa de bases de dados eletrônicas (MEDLINE, Embase, Cochrane, LILACS) e listas de referência de artigos.

**Seleção dos estudos** Selecionamos ensaios clínicos que avaliaram a influência das técnicas hemostáticas na reserva ovariana em pacientes com cistos ovarianos com aspecto ultrassonográfico benigno submetidos à ooforoplastia laparoscópica pela técnica de tração e contra-tração. Os estudos incluídos compararam as técnicas hemostáticas: sutura, energia bipolar, energia ultrassônica e selantes hemostáticos.

**Coleta de dados** Os desfechos avaliados foram o hormônio antimülleriano e a contagem de folículos antrais. A possibilidade de viés de publicação foi avaliada por gráficos de funil.

**Síntese dos dados** Doze estudos envolvendo 1.047 pacientes foram avaliados. A sutura foi superior à coagulação bipolar, e, na comparação entre selantes e energia bipolar, os resultados favoreceram o uso do primeiro grupo. O uso de energia ultrassônica não foi superior ao uso da energia bipolar.

**Conclusão** Em conclusão, recomendamos a sutura para hemostasia durante a ooforoplastia laparoscópica.

## Introduction

Ovarian cysts are a common gynecological situation, occurring in 6.6% of women between 25 and 40 years old.^1^ When its surgical removal is indicated, stripping the ovarian cyst wall by laparoscopic approach is the technique of choice.^2^ ^3^ However, surgical treatment may cause detrimental effects on ovarian reserve, which could occur because of removal of healthy ovarian tissue or by thermal damage to normal follicles during bleeding control.^4^

Ovarian reserve is marked as the size and quantity of the remaining ovarian follicular pool at any given time.^5^ It can be estimated by different methods, and the level of serum anti-Mullerian hormone (AMH) is considered one of the best endocrinologic marker.^6^ This hormone is a glycoprotein that is produced by the granulosa cells of the ovarian follicles, and it predicts the number of responsive follicles. The antral follicles count (AFC) may also be used, but it carries the inconvenience of only being able to be measured during a specific phase of the menstrual cycle.^7^

Bipolar electrocoagulation is the traditional hemostatic method for laparoscopic cystectomy. It is simple, fast and does not require advanced surgical skills. However, it may cause local thermal damage, compromising the ovarian reserve.^8^ In this scenario, laparoscopic suture is an interesting option, but it demands time to learn, master, and apply. An alternative method is the use of topical hemostatic agents, which induce clot formation.^9^

As the maintenance of a healthy and functioning ovarian tissue is prioritized during oophoroplasty, it is essential to estimate which hemostatic technique is less aggressive to the follicular reserve.

Therefore, the goal of this study as to analyze the impact on ovarian reserve (through the level of serum anti-Mullerian hormone and the antral follicle count) of the different hemostatic methods performed during laparoscopic cystectomy, using clinical trials.

## Methods

### Protocol and Registration

This systematic review was conducted in accordance with the preferred reporting items for systematic reviews and meta-analyses (PRISMA) recommendations.^10^ The review was registered on the PROSPERO international database (CRD42017060903).^11^

### Eligibility Criteria

- Types of studies - Clinical trials comparing different hemostatic methods in laparoscopic cystectomy. No publication date or language restriction was imposed for search strategy.
- Types of participants - Patients presenting ovarian cysts with benign sonographic appearance submitted to laparoscopic cystectomy by stripping technique.
- Types of intervention - We included trials that compared different laparoscopic hemostatic techniques: suture, bipolar electrocoagulation, ultrasonic energy and hemostatic sealants. Trials in which laparotomy was executed were excluded.
- Types of outcomes - Surgical impact on ovarian reserve was evaluated by the measurement of serum AMH level and AFC.

### Information Sources

Studies were identified by searching electronic databases (MEDLINE, Embase, Cochrane, LILACS) and scanning reference lists of articles. The gray and manual search was also performed through the analysis of theses, chapters of books, reference of references, guidelines and reviews.

### Search

The search strategy used for the MEDLINE and Embase databases was: (*ovarian cysts* OR ovarian cyst OR *teratoma* OR *teratomas* OR *dermoid cyst* OR *dermoid cysts* OR *endometrioma* OR *endometriomas* OR *endometriotic cyst* OR *endometriotic cysts* OR *non endometriotic cyst* OR *non endometriotic cysts*) AND (*stripping* OR *suturing* OR *suture* OR *bipolar* OR *electrocautery* OR *electrocoagulation* OR *coagulation* OR *sealant* OR *sealants* OR *hemostatic matrix* OR *hemostatic* OR *hemostatics* OR *hemostasis* OR *cystectomy*).” For the Cochrane and LILACS databases, the search strategy was: *cystectomy* AND *ovarian reserve*.

### Study Selection

Eligibility assessment and the selection of screened records were performed independently in an unblinded, standardized manner by two reviewers (Baracat C. M. F. and Bernardo W. M.). Disagreements between the reviewers were resolved by consensus.

### Data Collection Process

After the paper was read, we used a checklist based on the CONSORT recommendations for reporting a randomized clinical trial (http:// www.consort-statement.org/consort-statement). One review author (Baracat C. M. F.) extracted the data from each included study using a standardized form (Supplementary Information Sheet), and the second author (Bernardo W. M.) checked the extracted data.

### Data Items

Information was extracted from each trial on: (1) the characteristics of the trial participants, the characteristics of the cysts, and the trial's inclusion and exclusion criteria; (2) type of intervention and control groups (considering different hemostatic modalities: suture, bipolar electrocoagulation, ultrasonic energy and the application of hemostatic sealant); and (3) type of outcome measure (level of serum AMH and AFC).

### Risk of Bias in Individual Studies

Two reviewers worked independently and determined: the adequacy of randomization and concealment of allocation; the blinding of patients, healthcare providers, data collectors, and outcome assessors; and the correct report and extent of loss to follow-up. These items meet the criteria applied by the Jadad et al^12^ scale for the assessment of the risk of bias of randomized clinical trials; Jadad et al^12^ scores vary from 0 to 5 (scores lower than 3 indicate poor methodological quality), and they were calculated for each study.

At the study level, we also evaluated whether the hemostatic techniques were properly described (for example, the type of thread used to suture, the kind of suture, the power of electric current, the sealant used) or whether they were poorly stated or not described well enough to be reproduced. Besides these data, it was also analyzed whether the outcomes were well defined and detailed.

We did not intend to exclude any article from this review based on a higher risk of bias that it presented; however, it is important to point which studies present a higher methodological quality, especially if heterogeneous results appear.

### Summary Measures and Planned Methods of Analysis

Meta-analyses were performed by computing mean difference (MD), standard deviation (SD) and 95% confidence interval (CI) for each outcome, using fixed-effects model. If the results of a given article were expressed in minimum and maximum, the data were converted by the Hozo et al^13^ software. When the MD and SD of the decline in AMH levels were not available in the original papers, we calculated them from the published figures. Meta-analyses were conducted using the Review Manager 5.3 software, obtained from the Website of the Cochrane Informatics & Knowledge Management Department.^14^ The results were aggregated to meta-analyses using the inverse variance method to the continuous variables, and inconsistency (heterogeneity) was tested by the Chi-squared (Chi^2^) test and the Higgins et al^15^ method (I^2^).

### Risk of Bias across Studies and Additional Analyses

We assessed the possibility of publication bias by evaluating a funnel plot of the trials' mean differences for asymmetry. If the heterogeneity of the results of a meta-analysis (I^2^) was over 50%, we excluded the report(s) located outside the funnel (outliers) and then performed another meta-analysis without the given report. If we could not detect outliers, the random-effects model was chosen for the final result of the determinate meta-analyses, and true heterogeneity was presumed and discussed. We acknowledge that other factors could produce asymmetry in funnel plots, leading to a high heterogeneity (true study heterogeneity), such as differences in trial quality, differences in the population studied or likewise, differences in surgical skills.

## Results

### Study Selection

**Fig. 1:**
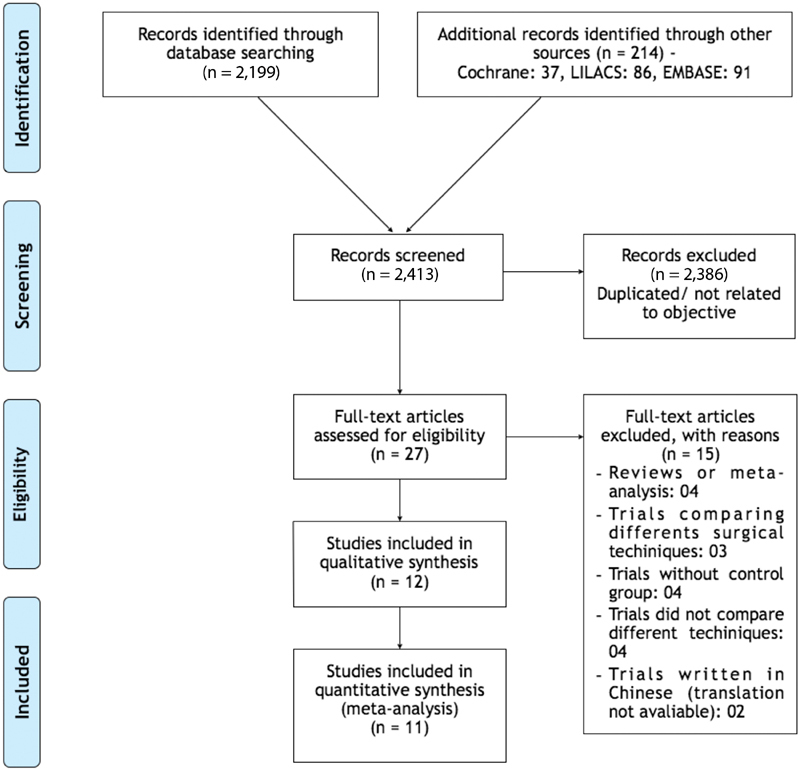
Study selection flowchart. Adapted from PRISMA.

Two thousand, four hundred and thirteen (2,413) studies were screened, and the articles were assessed for eligibility after the title and abstract were read. The following flowchart, an adapted PRISMA flow diagram, illustrates the study selection process (Fig. 1). One trial was excluded from the meta-analyses (Coric et al, 2011)^16^ because it measured the outcome in a different way (median of the sum of AFC in 3 postoperative cycles of operated ovaries) that could not be adapted to the outcomes reviewed in this paper.

### Study Characteristics

- Methods - Twelve clinical trials were included in the review, 10 of which were randomized and 2 that were non-randomized. All papers were published in English.
- Participants - A total of 1,047 patients were involved in this review. Although all patients presented ovarian cysts, some inclusion criteria were distinct between the trials, such as the etiology of the cyst. Most of the exclusion criteria were homogeneous between them (for example, previous use of hormones and endocrinopathy);
- Interventions - Each trial was placed under a specific comparison group according to the hemostatic techniques that were employed. One trial was placed under more than one comparison group, because it used 3 arms in the study, and each comparison was analyzed separately (Zhang et al, 2016).^17^ After the proper organization of the trials, we framed three comparison groups.: bipolar versus hemostatic agent, bipolar versus suture and bipolar versus ultrasonic energy. Each comparison group was meta-analyzed separately.
- Outcomes - Most of the trials assessed the same outcomes that we intended to evaluate. The studies measured the serum AMH and number of antral follicles at 1, 3, 6 and/or 12 months after surgery. Some studies expressed their results in decline rate of serum AMH (%), which was calculated as: 100 × ([preoperative AMH level - postoperative AMH level]/preoperative AMH level).

A summary of the characteristics of the included trials is shown in Table 1, organized into specific comparison groups.

**Table 1:** Characteristics of the included trials, organized into comparison groups

Author, year	n	Outcome measured	Measurement period	JADAD
**Bipolar x Hemostatic agent**				
Kang et al (2015)^18^	129	AMH	3 months	NR
Song et al (2014)^19^	100	AMH	3 months	3
Sönmezer et al (2013)^20^	30	AMH	1 and 3 months	3
**Bipolar x Suture**				
Zhang et al (2016)^17^	207	AMH and AFC	1, 3, 6, 12 months	2
Sahin et al (2017)^21^	90	AMH and AFC	1, 3, 12 months	3
Asgari et al (2016)^22^	109	AMH	3 months	3
Song et al (2015)^23^	125	AMH	3 months	NR
Tanprasertkul et al (2014)^24^	50	AMH	1, 3, 6 months	2
Özgönen et al (2013)^25^	60	AFC	1 e 3 month	2
Takashima et al (2013)^26^	44	AMH and AFC	3 months	1
Ferrero et al (2012)^27^	100	AMH	3, 6, 12 months	3
Coric et al (2011)^16^	50	AFC	2, 4, 6 months	3
**Bipolar x Ultrasonic energy**				
Zhang et al (2016)^17^	207	AMH and AFC	1, 3, 6, 12 months	2

Abbreviations: AFC, antral follicle count; AMH, anti-Mullerian hormone; n, number of patients; NR, not rated.

### Risk of Bias within Studies

The risk of bias within the studies was assessed by applying the Jadad et al^12^ scale and evaluating whether the hemostatic techniques and the definitions of the outcomes were properly described. No trial was double-blinded (as expected for this sort of trial), and this is considered a source of bias. Therefore, the maximum Jadad et al^12^ score of the articles was three points, which was achieved by the majority of the trials (Table 1).

Most of studies did not describe the hemostatic techniques used with much detail. When bipolar energy was used in the studies, there was heterogeneity in the power used by each author, ranging from 20 to 40 Watts; in addition, the duration of each energy pulse was not described by most studies. Regarding suture, the types of yarn and knot were also different among the tests, and most of them made use of polyfilament yarns. The application of the hemostatic agent was done following the technique recommended by each supplier, but only one study detailed the volume applied (Sönmezer et al, 2013).^20^

Regarding the outcomes, with the exception of one article, all studies that evaluated AMH levels described with accuracy the technical details of blood sample storage and the kit used for the enzyme-linked immunosorbent assay (ELISA), reiterating the importance of these particularities in the routine evaluation of this marker to avoid systematic errors. Most articles reported that the ultrasound examinations for the AFC were performed by the same evaluator.

Finally, in relation to accessory ports, two studies used laparoendoscopic single-site (LESS) surgery, while the other authors used two or three accessory trocars, but not all made clear the number of ports used.

### Results of Individual Studies

The serum AMH levels, the decline rate of serum AMH levels, and the number of antral follicles measured in each study are shown in Table 2.

**Table 2:** Number of antral follicles, serum anti-Mullerian hormone levels and decline rate of serum anti-Mullerian hormone measured in each study

Anti-Mullerian hormone								
Author, year	1m	3m	6m	12m	1m	3m	6m	12m
	Bipolar energy				Suture			
Sahin et al (2017)^21^	2.32 ± 2.01	2.38 ± 2.57	−	2.78 ± 2.85	3.24 ± 3.01	3.17 ± 3.40	−	3.71 ± 3.09
Zhang et al (2016)^17^	1.90 ± 0.70	1.80 ± 1.0	1.90 ± 0.80	2.0 ± 0.90	2.90 ± 1.80	3.0 ± 1.8	3.0 ± 1.5	3.1 ± 1.6
Asgari et al (2016)^22^	−	1.25 ± 0.84	−	−	−	2.10 ± 0.88	−	−
Tanprasertkul et al (2014)^24^	1.76 ± 1.50	2.09 ± 1.66	2.11 ± 1.84	−	2.09 ± 1.62	1.96 ± 1.68	1.72 ± 1.68	−
Takashima et al (2013)^26^	−	3.16 ± 1.27	−	−	−	2.88 ± 0.83	−	−
Ferrero et al (2012)^27^	−	1.85 ± 1.67	1.75 ± 1.94	1.75 ± 1.82	−	2.65 ± 2.42	2.25 ± 1.53	2.30 ± 1.77
	**Bipolar energy**				**Hemostatic agent**			
Song et al (2014)^19^	−	2.04 ± 0.64	−	−	−	2.67 ± 0.95	−	−
Sönmezer et al (2013)^20^	1.64 ± 0.93	2.84 ± 1.12	−	−	2.72 ± 1.49	3.07 ± 1.43	−	−
	**Bipolar energy**				**Ultrasonic energy**			
Zhang et al (2016)^17^	1.9 ± 0.7	1.8 ± 1.0	1.9 ± 0.8	2.0 ± 0.9	1.5 ± 0.9	1.8 ± 0.9	1.9 ± 1.0	2.0 ± 1.0
**Decline rate of serum AMH**								
	**Bipolar energy**				**Suture**			
Asgari et al (2016)^22^	53.42 ± 15.28				15.94 ± 18.55			
Song et al (2015)^23^	42.2 ± 9.12				24.6 ± 6.35			
	**Bipolar energy**				**Hemostatic agent**			
Kang et al (2015)^18^	41.2 ± 8.92				15.4 ± 6.1			
Song et al (2014)^19^	41.2 ± 9.32				16.1 ± 9.1			
**Number of antral follicles**								
**Author, year**	**1m**	**3m**	**6m**	**12m**	**1m**	**3m**	**6m**	**12m**
	**Bipolar energy**				**Suture**			
Zhang et al (2016)^17^	3.2 ± 1.6	3.6 ± 1.3	3.9 ± 1.4	4.2 ± 1.5	3.1 ± 1.4	4.7 ± 1.3	6.0 ± 1.9	6.3 ± 2.0
Sahin et al (2017)^21^	4.26 ± 4.12	5.30 ± 4.59	−	5.86 ± 4.53	6.79 ± 4.54	7.48 ± 5.02	−	7.55 ± 5.23
Takashima et al (2013)^26^	−	2.7 ± 0.9	−	−	−	1.7 ± 0.7	−	−
Özgönen et al (2013)^25^	6.9 ± 1.04	8.63 ± 1.21	−	−	7.23 ± 1.38	8.60 ± 1.24	−	−
	**Bipolar energy**				**Ultrasonic energy**			
Zhang et al (2016)^17^	3.2 ± 1.6	3.6 ± 1.3	3.9 ± 1.4	4.2 ± 1.5	3.0 ± 1.7	3.6 ± 1.4	3.9 ± 1.3	4.0 ± 1.2

Abbreviations: AMH, anti-Mullerian hormone; m, months; -, not rated.

### Synthesis of Results and Risk of Bias across Studies

In the following figures, AMH or AFC means and their standard deviations, and the results of the meta-analyses (mean differences) and their respective heterogeneity measures are graphically exhibited; this was performed for every comparison group. We developed one forest plot and one funnel plot for each outcome, and an additional forest plot excluding the outliers, if necessary. As mentioned before, if heterogeneity kept high, the random-effects model was applied. Due to the great amount of graphics, only the most important ones will be shown here.

### Bipolar versus Hemostatic Agent

This comparison group contains three trials to be meta-analyzed, all of them measured serum AMH at the 3^rd^ month after surgery. However, two expressed their results as decline rate of serum AMH level as we already described.

Decline rate of serum AMH level - As shown in Fig. 2, the results of this meta-analysis favored the use of hemostatic agents, when compared the different types of sealants jointly and isolated (MD = 25.52, CI 95% = 23.23–27.81 and MD = 25.44, CI 95% = 22.83–28.04 respectively), both analysis without heterogeneity (I^2^= 0%).

**Fig:**
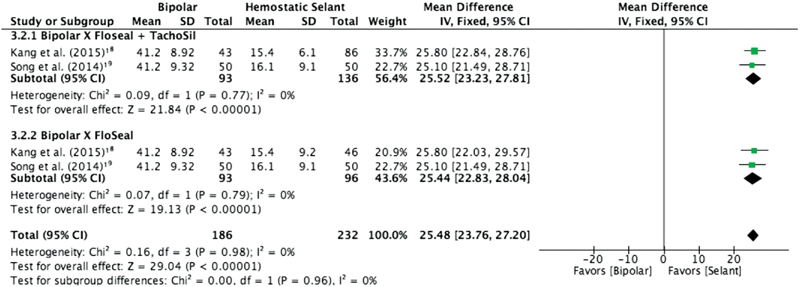
. **2** Decline rate of serum anti-Mullerian hormone 3 months after surgery: bipolar versus hemostatic agents.

Serum AMH level at the 3rd month after surgery - There were statistically significant differences in this hormone level, favoring hemostatic agents (MD = -0.59, CI 95% = -0.89–-0.29), with I^2 ^= 0%.

### Bipolar versus Suture

In this comparison group, eight trials were included, three of them measured serum AMH level and four measured only ACF. In this last group, one study measured the outcome in a different way (median of the sum of AFC in three postoperative cycles of operated ovaries) and because of that, we did not include it in the meta-analyses (Coric et al, 2011).^16^

Decline rate of serum AMH level - Despite the high heterogeneity (I^2 ^= 96%), this meta-analysis favored suture (MD = 20.30, CI 95% = 17.73–22.86). We applied the random-effect model to neutralize sample size, and the result was sustained (MD = 27.27, CI 95% = 7.80–46.75).

Serum AMH level at the 1^st^, 3^rd^, 6^th^ and 12^th^ month after surgery - When evaluating the hormone level in the 1^st^ month postsurgery, the meta-analysis showed a statistical difference in comparison between suture and bipolar, favoring suture (MD = -0.86, IC95%= -1.25–-0.47), with no heterogeneity.

**Fig. 3:**
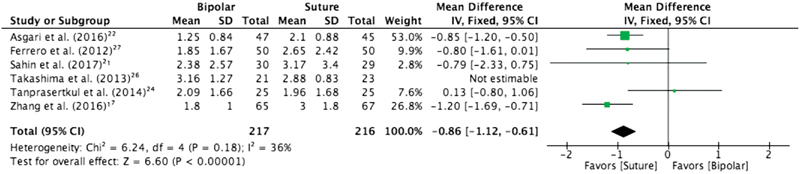
Serum anti-Mullerian hormone 3 months after surgery: bipolar versus suture, excluding the outlier.

The analysis at the 3^rd^ month postsurgery was the comparison that contained the greatest amount of trials, with a total of 6 studies. The result was significantly favorable to suture (MD = -0.70, CI 95%= -0.94–-0.47), with a high heterogeneity between trials, I^2^= 70%. After applying the sensitivity test through the funnel plot, we identified one outlier (Takashima et al., 2013).^26^ The heterogeneity of the analysis dropped to 36% after removing this outlier, maintaining statistical difference favoring the suture group (MD = -0.86, CI 95%= -1.12–-0.61), as shown in Fig. 3.

At 6 months postsurgery, the results favored suture again (MD = -0.79, CI 95%= -1.12–-0.45), with I^2^= 76%. The trial conducted by Tanprasertkul et al (2014)^24^ was considered as an outlier in the funnel plot. After the removal of this trial, the heterogeneity dropped to I^2^= 54%, showing statistical differences to the use of suture (MD = -0.94, IC95% = -1.29–-0.59).

Not coincidentally, those two studies pointed as outliers in the previous meta-analyses presented lower methodological quality at the risk of bias evaluation. The trial conducted by Takashima et al^26^ scored 1 point according to the Jadad et al^12^ scale and the one by Tanprasertkul et al (2014)^24^ scored 2 points.

**Fig. 4:**
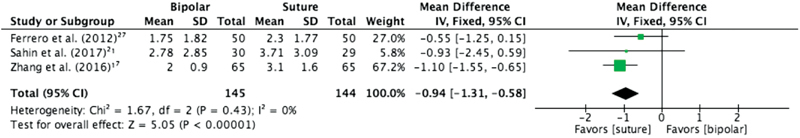
Serum anti-Mullerian hormone 12 months after surgery: bipolar versus suture.

Concordant to the others results, meta-analysis at 12 months after surgery appointed suture as the best hemostatic technique, with homogeneous results (MD = -0.94, IC95% = -1.31–-0.58) and no heterogeneity (I^2 ^= 0%) (Fig. 4).

AFC - To evaluate this outcome, we got clinical trials for the meta-analysis only at 1, 3 and 12 months after surgery.

In the 1^st^ meta-analyses, 1 month after surgery, there was no difference between bipolar and suture (MD = -0.15, CI 95% = -0.54–-0.24); however, with high heterogeneity (I^2 ^= 65%). We conducted the sensitivity tests through a funnel plot, which showed Sahin et al (2017)^21^ as an outlier. Heterogeneity dropped significantly (I^2 ^= 9%) after removal of this trial, showing no statistical differences between bipolar and suture.

The AFC at 3 months postsurgery identified a statistical significance favoring suture (MD = -0.75, CI 95% = -1.11–-0.39), with high heterogeneity once more (I^2 ^= 80%). Therefore, we considered the funnel plot and excluded the trial by Özgönen et al^25^ (as an outlier), and suture was favored (MD = -1.13, CI95% = -1.57–-0.70), with no heterogeneity.

**Fig. 5:**
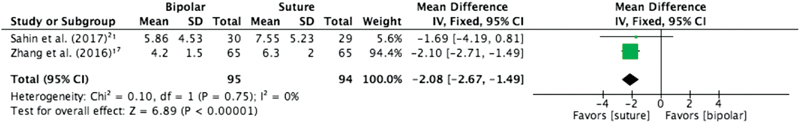
Antral follicle count 12 months after surgery: bipolar versus suture.

After 1 year postsurgery, the effect on ovarian reserve was sustained, and suture was less harmful to AFC than bipolar (MD = -2.08, IC 95% = -2.67–-1.49) with I^2 ^= 0% (Fig. 5).

### Bipolar versus Ultrasonic Energy

There was only one trial included in this group, and it measured serum AMH level at the 1^st^, 3^rd^, 6^th^ and 12^th^ month postsurgery. In the first analysis, there was a statistical difference favoring bipolar energy (MD = 0.40, IC95% = 0.12–0.68), while in the subsequent months, no difference between energies was detected (MD = 0.00).

## Discussion

We consider that this systematic review involved a substantial number of trials, most of which were considered as presenting a high methodological quality, and it evaluated solid and relevant outcomes in different periods after the surgical procedure. Those facts brought some very interesting results that deserve to be discussed.

Bipolar electrocoagulation was considered the control group in all studies, as it is widely performed, most likely because of its ease of use, availability, and the extensive experience with its application. However, the results show that the use of other hemostatic techniques minimizes the surgical impact on ovarian reserve.

In the comparison group that confronted suture with bipolar energy, the evaluation of ovarian reserve was favorable to the suture technique in practically all the analyses. When the AMH level was measured, the analysis performed at 3 months postoperatively (group with the highest number of included studies) presented a high heterogeneity, which was solved after the sensitivity test by means of the funnel plot. It pointed out Takashima et al (2013)^26^ as the outlier, emphasizing that this study was only included in this analysis. We believe that its discrepancy was due to the use of vasopressin for dissection of ovarian capsule, since this was the only trial to use this technique. Moreover, this was the only study that scored 1 point in the Jadad et al^12^ scale, suggesting a low methodological quality. At the 6^th^ month of evaluation, the result also favored suture, with high heterogeneity again; however, this time, it was attributed to the Tanprasertkul et al (2014).^24^ This study did not feature any peculiarity in its surgical technique or in the selection of patients; meanwhile, it was also considered a trial of low methodological quality. In the analysis after 12 months of the surgery, we found that the suture was favored with statistical significance and low heterogeneity, denoting the possibility of a long-term benefit for this technique.

In relation to AFC, the results were similar. In the evaluation at 3 months postsurgery, the analysis showed high heterogeneity and the sensitivity analysis pointed Özgönen et al^25^ as the discrepant study, once more a trial with poor methodological quality. After its exclusion and reanalysis, the heterogeneity dropped to zero. The evaluation at the 12^th^ postoperative month had no heterogeneity, stablishing the long-term superiority of the laparoscopic suture technique in the maintenance of ovarian reserve. These consistent results serve as an incentive for surgeons to master this laparoscopic technique.

The results generated by the meta-analyses of the comparison between bipolar electrocoagulation and the application of hemostatic sealants were favorable to the second group. It is worth mentioning that the only outcome evaluated was the AMH level in the 3^rd^ postoperative month, measured in its absolute value and in the difference of the AMH levels. Although only two studies were included in each of these analyses, the heterogeneity was null and the subgroup analysis with the different types of sealants did not favor a specific agent. This analysis shows that probably the lesser tissue manipulation that the sealant promotes interferes positively in the prognosis of ovarian reserve. The disadvantage of these agents is the possibility of FloSeal to trigger an allergic reaction and formation of an eosinophilic granulomatous tissue,^28^ ^29^ or intra-abdominal adhesions,^28^ ^30^ ^31^ besides their high cost.

Only one study compared bipolar energy versus ultrasonic energy and its results were not statistically relevant. The scarcity of studies in the literature involving this type of energy is expected if we consider that ultrasonic energy has other primordial utilities. However, as there are situations in which cystectomy is performed along with the resection of endometriosis foci, in which the ultrasonic energy has good applicability, it would be interesting to know if the use of this energy is beneficial. Therefore, new studies may add to this analysis for more robust results.

We believe that other technical factors are also important for the maintenance of a healthy ovarian parenchyma. One of them would be the number and location of the accessory points, which varied enormously in most articles. In addition, the studies did not mention the duration of application of bipolar energy in each region or the number of times it was applied. Regarding suture use, the trials varied in the type of yarn and number of stitches applied. As for the sealant, only one study reported the area covered by the product. Thus, it is difficult to reach any solid conclusion regarding these variables on ovarian reserve.

One limitation of this systematic review is the relatively small number of studies involved in each meta-analysis. We also believe that more studies with a longer time to follow the patients are necessary to determine more accurately the long-term advantages of each method. It is important to consider that any systematic review includes heterogeneous patients. That heterogeneity is minimized by sensitivity analyses and risk assessments of bias. However, its results should not be considered as absolute.

## Conclusion

In conclusion, the results of this systematic review show that suture is superior to bipolar electrocoagulation when considering ovarian reserve after laparoscopic cystectomy. Moreover, this superiority appears to be sustained at long-term follow-up. Hemostatic sealants application has demonstrated better preservation of the ovarian follicles than bipolar electrocoagulation at the 3^rd^ postoperative month. There are no trials evaluating long-term outcomes. The use of ultrasonic energy seems to cause similar damage to ovarian reserve when compared with bipolar energy. Based on what has been presented by this systematic review, we recommend the application of suture to achieve hemostasis in laparoscopic cystectomy.
